# Over a century of cancer research: Inconvenient truths and promising leads

**DOI:** 10.1371/journal.pbio.3000670

**Published:** 2020-04-01

**Authors:** Carlos Sonnenschein, Ana M. Soto

**Affiliations:** 1 Tufts University School of Medicine, Boston, Massachusetts, United States of America; 2 Centre Cavaillès, Ecole Normale Supérieure, Paris, France

## Abstract

Despite over a century of intensive efforts, the great gains promised by the War on Cancer nearly 50 years ago have not materialized. Since 1999, we have analyzed the lack of progress in explaining and “curing” cancer by examining the merits of the premises that determine how cancer is understood and treated. Our ongoing critical analyses have aimed at clarifying the sources of misunderstandings at the root of the cancer puzzle while providing a plausible and comprehensive biomedical perspective as well as a new theory of carcinogenesis that is compatible with evolutionary theory. In this essay, we explain how this new theory, the tissue organization field theory (TOFT), can help chart a path to progress for cancer researchers by explaining features of cancer that remain unexplainable from the perspective of the still hegemonic somatic mutation theory (SMT) and its variants. Of equal significance, the premises underlying the TOFT offer new perspectives on basic biological phenomena.

## Abstract 2

This Essay proposes that a new theory of cancer — the “tissue organization field” theory — explains phenomena (such as the normalization of cancer cells) that are not consistent with the widely held somatic mutation theory.

“About 30 years ago there was much talk that Geologists ought only to observe & not theorise; & I well remember some one saying, that at this rate a man might as well go into a gravel-pit & count the pebbles & describe their colours. How odd it is that every one should not see that all observation must be for or against some view, if it is to be of any service.”—*Charles Darwin letter to Henry Fawcett*. *18 September 1861*.: *The correspondence of Charles Darwin*. *Edited by Frederick Burkhardt et al*. *Cambridge*: *Cambridge University Press*. *1985*.

## Introduction

The lack of significant improvements in the understanding of carcinogenesis, and the failure to reach the cherished goal of “curing” cancer as envisioned by the 1971 War on Cancer declaration, encouraged several researchers to question the strategy of the war effort during the last quarter century [1–7]. The consensus reached by critics centered on the recognition that advances in understanding what happens within cells (in nuclei, mitochondria, endoplasmic reticulum, cell metabolism, plasma membrane, etc.) remained mostly irrelevant both to understanding carcinogenesis and to significantly benefiting the object of the whole effort, the cancer patient [8,9]. As a result, researchers, clinicians, and patients have called for a critical evaluation of theories of carcinogenesis [8,10–24].

In this Essay, we address these issues from a historical perspective, outline the merits of the 2 main theories of carcinogenesis, and draw—when possible—constructive conclusions. Given the vastness of the topic, we are necessarily leaving out a few historical milestones [6,25–28].

## Background

During the second half of the 19th century, cancer was considered a tissue-based disease, and proliferation was a constitutive property of cells. In 1914, the famed embryologist/zoologist Theodor Boveri, who admitted to not having hands-on experience in carcinogenesis, stated that the initial steps of the carcinogenic process (initiation and early progression), unlike those of the embryos he studied, were not accessible to observers because tumors had already accumulated billions of cells by the time they were detected [29]. Therefore, it was not possible to accurately describe how a palpable tumor initially arose. This conclusion remains unaltered today. Also, Boveri agreed—as did his predecessors—that the default state of cells was proliferation, but in opposition to the then prevailing view, he posited that cancer was a cell-based disease due to chromatin changes (mutations) that modified the proliferative behavior of what he considered to be “the cancer cell” [30]. Over the years, alternative conjectures and hypotheses about cancer initiation have been proposed by scientists who, in some cases, like Boveri, had no first-hand experience on the subject yet freely speculated about it [31–34], while others expounded their empirical-based views on carcinogenesis [6,35–40].

In addition to Boveri’s remarks on cancer initiation’s observability, other factors have contributed to obfuscation in this field. Namely, since the beginning of the 20th century, leading cell and developmental biologists who once considered theories necessary to understand the objects of inquiry adopted, instead, the belief that reality is directly accessible by the accumulation of data, which made theories superfluous [41–43]. Concomitantly, they embraced a reductionist stance [44]. Evolutionary biologists, on the other hand, continued to appreciate theory’s practical benefits in framing observations and experiments while incorporating new discoveries that did not fit the current version of Darwin’s theory [45–47] along with new and updated theoretical perspectives [48]. This updating is possible because evolution’s principles have been clearly stated, i.e., it is not a vague theory (see below). In contrast, experimental biologists dealing with aspects of the life cycle of organisms distanced themselves even more from theoretical concerns during the second half of the 20th century as the molecular biology revolution took off. Indeed, molecular biologists radically modified the way organisms were thought about by introducing mathematical information theories into biology concepts without a proper analysis while using metaphors that were eventually taken literally [49]. By the 1950s, the standard view of carcinogenesis became dominated by the somatic mutation theory (SMT), which is rooted in a reductionist and genetic deterministic stance, including the erroneous assumption of a linear causal link between genotype and phenotype [50]. This stance predicates that cells are like tiny computers run by a program [51–58]. The lack of a robust theoretical background to frame experiments and explanations has hindered the adoption of productive alternatives in the fields of experimental biology and cancer research for decades [22,59–61].

## Evaluating theories of carcinogenesis

We will first compare the merits of what we consider the 2 main theories of carcinogenesis, namely, the still hegemonic SMT and the more recently proposed tissue organization field theory (TOFT) [6] (see Table 1). The rationale for reducing the number of theories on carcinogenesis to 2 is due to the fact that they can be distinctively separated by their alleged place of origin within the host organism and by the different roles that mutations play in both theories. In the latter regard, mutations are central for the SMT, whereas they are considered as an epiphenomenon by the TOFT.

**Table 1:** Comparison between the SMT and the TOFT.

	SMT	TOFT
**Premise 1:** What is the default state of cells in multicellular organisms?	Proliferative quiescence. Cells require stimulation by external (“growth factors”) and/or intrinsic (“oncogenes”) factors.	Proliferation with variation and motility. Regulation of constitutive cell proliferation and of motility is exclusively exerted by external and/or intrinsic inhibitors of these functions.
**Premise 2:** How does the process of carcinogenesis take place?	Changes in the DNA of the founder cell makes this cell unable to control its proliferation. As a consequence, a neoplasm will be formed.	Carcinogenesis is “development gone awry.” Chronic abnormal interactions between mesenchyme/stroma and parenchyma of a given morphogenetic field are responsible for the appearance of a neoplasm.
Level of biological organization at which carcinogenesis takes place	Cellular	Tissue
Target disrupted by the carcinogenic insult	DNA	Morphogenetic field
Role of DNA mutations	Causal	Irrelevant, epiphenomenon
Consequence of the insult	1. Uncontrolled cell proliferation. 2. Formation of a clonal neoplasm in which all cells are mutated in the same gene(s) affecting the control of cell proliferation. 3. Additional mutations are invoked by most researchers to explain metastasis.	1. Altered tissue structure involving hyperplasia, metaplasia, dysplasia, and carcinoma. 2. Formation of polyclonal neoplasms. 3. The constraints imposed by the tissue to its cells are impaired. As a result, cells express their default state (i.e., proliferate and migrate), thus causing tumor growth, invasion, and metastases.
Weaknesses and strengths of theories of carcinogenesis	1. Failure in explaining “foreign-body” carcinogenesis due to a lack of induced DNA mutations by physical or inert materials. 2. Failure in explaining the normalization/”maturation” of cancer tissues when they undergo “spontaneous regression.” 3. In principle, no objection in explaining germline cancers by DNA mutations that may alter the control of cell proliferation.	1. Explains “foreign-body” carcinogenesis as an unspecific tissue disruption of a morphogenetic field. 2. “Spontaneous cancer regression” is compatible with the TOFT. Tissue recombinants show that cancer cells (even of those carrying alleged “oncogenic” mutations) are “normalized” when placed in homotypic “normal” tissues. 3. The TOFT explains germline cancers through a morphogenetic field effect because the mutation is present in all cells in the affected organism.
Corollary	Irreversibility. “Once a cancer cell, always a cancer cell.”	Reversibility. Due to spontaneous and induced normalization, cancer is not destiny.

**Abbreviations:** SMT, somatic mutation theory; TOFT, tissue organization field theory

The SMT is anchored at the cellular level of biological organization, which has theoretical and experimental implications. If “a cell” is the crucial target of carcinogens, the research strategy to be followed highlights events to be found inside cells. Adequate models for this strategy can then be adopted as exemplified by experiments using a single cell type in a 2D cell culture. In contrast, the TOFT, which is anchored at the tissue level of biological organization, explores carcinogenesis as a relational problem whereby the reciprocal interactions among the diverse cell types of the morphogenetic field take place. The TOFT focuses not on a single cell type but, as in organogenesis, on the interactions among different cell types and compartments. The difference between these theories can be summarized as “the renegade cell” of the SMT [62] versus “development gone awry” of the TOFT [63].

Equally important, if not more so, these 2 theories adopt distinctive premises regarding inherent cell behavior. That is, the cell-centered theories implicitly adopt the premise that quiescence is the constitutive (default) state of cells in multicellular organisms, and therefore cells require intrinsic or extrinsic stimulation by either “oncogenes” or “growth factors,” respectively, in order to proliferate [36,54]. In contrast, the TOFT posits that proliferation is the default state of all cells, and therefore only inhibitory constraints prevent them from entering the cell cycle [6,39]. Thus, according to the TOFT, cells constitutively proliferate and generate movement, and the tissues in which they reside constrain their ability to proliferate and move. When a carcinogen loosens the tissue constraints, cells regain their default state and proliferate again, eventually forming a tumor mass, also allowing cells to move, invade tissues, and metastasize. Therefore, the relevant question is not what drives proliferation and motility but what tissue constraints inhibit both processes and how they operate in the altered morphogenetic field.

In light of the SMT’s failure to explain carcinogenesis and deliver promised therapeutic successes, a third group of cancer theories has been introduced that combines a cell-based theory with elements of the tissue-based theory to explain the initial steps of carcinogenesis [36,37]. In essence, this latter group of theories retain the core of the SMT while adding the microenvironment in the form of the multiple cell types that surround the single initiating “cancer cell” [64–68]. They claim that either the cancer cell and its descendants “recruit” the cells in their microenvironment to “prosper” or that perturbations in the stroma induce DNA mutations and translocations in the parenchymal cells [69–71]. In short, this latter group of theories is proposed as a sort of compromise between the SMT and the TOFT [15,68,72–75]. In reality, they are variants of the SMT, given that somatic mutations still play a causal role in them (Table 1). These SMT theories may have generated confusion about a role of mutations in the TOFT; the central idea of the SMT, that DNA mutations are causal, is so ingrained that some authors have taken it for granted that the TOFT also claims a causal role for mutations [76], even though the TOFT has always considered DNA mutations an epiphenomenon.

## A sequence of misperceptions in biology

The resolution of the controversy about theories of carcinogenesis is not an inconsequential “academic” pursuit but directly impacts glaring inconsistencies that arise when interpreting data generated under the premises of the SMT. More significantly, resolving this controversy will help correct misunderstandings about basic biological phenomena such as the default state of cells and causality among levels of biological organization. In addition, the resolution of this controversy will provide guidance in medically related issues such as cancer diagnosis and treatment [6].

Adoption of the cell-centered theory of carcinogenesis, i.e., the SMT and its variants, has led to several misconceptions. According to the SMT, somatic DNA mutations in cancer cells induce them to proliferate incessantly; this is not supported by evidence given that those cells can either proliferate or be dormant [77,78]. Moreover, they do not proliferate autonomously, as exemplified by their fate in hormone-dependent cancers [79]. The implicit premise that the constitutive (default) state of cells in multicellular organisms is quiescence generated the search, identification, and characterization of putative growth factors [80] and oncogenes [81–85]. Paradoxically, however, conclusions that those putative growth factors and oncogenes are indeed direct stimulators of cell proliferation have been duly contradicted by evidence and acknowledged even by those who originated this significant misperception [6,80–87]. Given that proliferation is considered the noncontroversial default state of unicellular organisms [54, 88], a switch from proliferation as the default state in unicellular organisms to quiescence in multicellular ones is an evolutionary novelty that should have been noted, highlighted, and explained. Curiously, notwithstanding, the current dominant perception in the field of biology at large that the proliferation of cells in multicellular organisms depends on being directly stimulated by “growth factors” and/or “oncogenes” remains practically unaltered. A clarification of this basic biological principle is highly overdue through a rigorous analysis of the published record. Next, the subsequent adoption of evolutionarily relevant premises that replace the unreliable ones should be implemented (proliferation as the default state of all cells and a search and characterization of inhibitors of cell proliferation), as we have argued previously [6,28,89–91]. Altogether, the unchallenged adoption of the misleading premises of the SMT gave way to the primacy of a strict reductionist approach to the subject of carcinogenesis.

For decades, the management of clinical cancers has been based on the premises of the SMT that, in short, meant to kill the allegedly immortalized, mutated “cancer cells.” This approach ignores evidence that the carcinogenic process is reversible, as repeatedly proven both experimentally [92–95] and clinically [14,16,38,96]. Regardless of these damning conclusions, the SMT and its variants have maintained their hegemony in academic circles, in the hospital ward, in BigPharma, in the specialized and lay media at large, and, equally important, in study sections of funding agencies, in which short- and long-term future trends in cancer research are decided.

## Examining explicit and implicit premises

Theories and their principles not only provide likely explanations of phenomena but also play a central role in determining what can be observed and in framing experiments. Thus, experiments can be designed to simultaneously test alternative theories, such as the TOFT and the SMT, to explain mammary gland carcinogenesis after exposure to a chemical carcinogen. Whereas the TOFT posits that the target of carcinogens is the reciprocal interactions between tissues (stroma and epithelium) and their cells, the SMT, in contrast, claims that carcinogens directly cause cancer by inducing mutations in the cells that will become “cancer cells” (in this case, the epithelial cells). In order to simultaneously test both theories, we separately exposed the mammary gland epithelium and stroma to carcinogens and then recombined these tissues to identify the target of the carcinogen (either the epithelium or the stroma or both components of the morphogenetic field). These experiments revealed that, as expected from the TOFT, the stroma is the target of the carcinogen and showed that unexposed epithelial cells acquired neoplastic properties. This experimental design could not have been conceived when adopting the premises of the SMT, for which the direct target is an epithelial cell [97].

SMT explanations of carcinogenesis remain narrowly framed at a molecular level, centered on mutated genes and their altered coded proteins [1,36,57,98,99]. That narrow focus, along with the acceptance of metaphors for information, program, and signal as real entities, has led to the erroneous assumption that there is no need to define the theoretical framework for the experimental design and the interpretation of data on carcinogenesis. In contrast, the TOFT is rooted in 3 fundamental, interdependent biological principles explicitly proposed for the construction of a Theory of Organisms [100]: (a) a principle of biological inertia, i.e., the default state (proliferation with variation and motility) [6,91]; (b) the principle of variation, which applies both at the cellular and supracellular levels, generating novelty [101]; and, finally, (c) the principle of organization, which deals with the generation and maintenance of stability by the closure of constraints [17,102].

Central to our Theory of Organisms lies a fundamental biological concept: the cell theory. According to Darwin’s theory of evolution, all living organisms originated from a common ancestor that must have been endowed with the capacity to proliferate constitutively, as long as conditions for survival were met. As multicellularity emerged [103], sexually reproducing multicellular organisms developed from a unicellular entity, i.e., the zygote [91,100,104]; moreover, development proceeds under theoretical principles that govern cell behavior. The default state entails that cells are normative agents that initiate actions, including proliferation and motility, which figure prominently in the process of carcinogenesis and metastasis. In addition, because a cell generates 2 slightly different daughter cells, proliferation produces variation [91]. Again, the principle of variation applies to the incessant changes that occur throughout the life span of organisms and generate individuation and novelty [105]. Also, the principle of organization addresses the stability of organisms by the interdependence of vital processes, technically referred to as “closure of constraints” [106]. These principles provide a testable framework whereby normal development and its alterations, including carcinogenesis, can be conceptually understood, experimentally explored, and mathematically modeled [100,101,107].

## Differing theoretical frames of the SMT and TOFT

One reason the field of cancer theories has remained controversial, in our view, relates to the terms used under the 2 main frameworks. For instance, the meaning of the terms “cell-centered” and “tissue-centered” ought to be spelled out in a clinical context. As claimed by the SMT, carcinogenesis is initiated in a single cell, namely, an epithelial cell in carcinomas, which represent 90% of clinical cancers. From the original simple claim that a single mutated gene (an oncogene) originated a cancer, the SMT was amended repeatedly when evidence failed to fit theory. For example, from a single dominant oncogene, there are now more than 300 “driver” genes. Later, the notion of antioncogenes (or suppressor genes) was introduced [108]. Finally, as mentioned above, the SMT was further extended by invoking elements of the TOFT while retaining the original mutated cell as crucial to the carcinogenic process.

As more new elements are added to the SMT to accommodate the lack of fit between the original theory and evidence, the vaguer the theory becomes. As Richard Feynman stated, a vague theory cannot be proven wrong [109]. Indeed, the original version of the SMT could have been proven wrong early on when it was shown that one so-called oncogene did not cause cancer. However, instead of a critical analysis and eventual rejection, criticisms were ignored, and the theory was temporarily rescued by adding more and more elements.

Under the cell-centered SMT narrative, “the cancer cell” in humans eventually “progresses” (through iterative proliferation) after either weeks, months, years, or decades to become a palpable, primary tumor and to metastasize. Under the tissue-centered TOFT, the initial carcinogenic event that generates the palpable primary tumor mass takes place at the tissue level of biological organization in which the interaction among multiple epithelial cells and adjacent stromal components (i.e., the morphogenetic field) has been broken and is being repaired [6,17,39,110]. It is not the timetable required for a tumor mass to be noted that differs in these theories but the protagonists of the process—the cell for the SMT and the morphogenetic field for the TOFT. In essence, the genomic mutations alleged to be causal for the SMT are irreversible and so should be the cancers formed from them, whereas the perturbations of the morphogenetic field are reversible (“development gone awry”) and so would be their cancers (see Table 1).

The classification of neoplasms according to when tumors appear during the host’s lifetime remains mostly intact in the TOFT. That is, “sporadic” cancers are those diagnosed in adults (about 95% of clinical cancers), while what we have called “inborn errors of development” occur in just about less than 5% of cancer patients [111]. In turn, the “inherited” variety of these inborn errors of development are cancers that mostly appear early in life (in fetuses, infants, and young adults). In carriers of germline mutations (less than 2% of all detected cancers), all the cells of these young patients carry these mutations, and therefore all the cells in the affected morphogenetic field (epithelium/parenchyma and stroma) are actively involved in the carcinogenic process.

## Corollaries and predictions

Theories determine what can be observed and thus the type of experiments that could be performed within the frame proposed by the theory. If the SMT is adopted, one will look inside cells and search for DNA mutations and stimulatory factors that would enhance cell proliferation, motility, and thus invasion and the ability to metastasize. From the SMT perspective, mutational events are hardwired, and cancer is considered irreversible. However, this theoretical framework is challenged by an increasing number of publications showing the presence of mutated oncogenes [112] and of aneuploidy in normal tissues [113]. Coupled with the existence of tumors containing neither gene mutations nor epigenetic aberrations [114], these findings show that somatic mutations are neither necessary nor sufficient for cancer to develop [16]. A corollary of this view is that “the cancer cell” does not exist, per se [77].

These conceptual differences result in different predictions and outcomes. Cancer normalization is explained by the TOFT but not by the SMT. Spontaneous cancer regression is due not only to the death of cells in a tumor but also to the remodeling of the cancerous tissue and the consequent “normalization” of the cells inside it. This normalization phenomenon has been documented in neuroblastomas in which the Schwann cells of the stroma are thought to induce the maturation of proliferating neuroblasts into mature, nonproliferating ganglion cells [115]. Moreover, conclusions drawn from the original experiments by Mintz and Illmensee in the 1970s and others since then buttress the notion that normalization is experimentally reproducible [94,116,117] and that it also occurs in clinical settings.

## Conclusions

For the sake of the current and future credibility in the fields of biology at large and cancer in particular, a rigorous, critical assessment of the odd situation biologists and cancer researchers are facing is in order. Our long-term analysis of the theoretical bases under which cancer research has been and is conducted, and the empirical evidence collected after more than a century of intensive research, indicates that the reductionist SMT should be abandoned and replaced by an organicist theory that adopts reliable principles relevant to the theories of evolution and organisms [118]. Switching theories will have profound effects on biology at large and, critically, on how researchers frame experiments and interpret observations in normalcy and cancer.

## Abbreviations

SMT: somatic mutation theory
TOFT: tissue organization field theory
